# Autonomous and non-autonomous roles of DNase II during cell death in *C. elegans* embryos

**DOI:** 10.1042/BSR20150055

**Published:** 2015-06-11

**Authors:** Hsiang Yu, Huey-Jen Lai, Tai-Wei Lin, Szecheng J. Lo

**Affiliations:** * Department and Graduate Institute of Biomedical Sciences, College of Medicine, Chang Gung University, Taoyuan 33302, Taiwan; † Department of Molecular, Cellular and Developmental Biology, University of Colorado, Boulder 80302, U.S.A.

**Keywords:** apoptosis, *Caenorhabditis elegans*, DNase II, exocytosis, fluorescence probes, lysosome

## Abstract

Generation of DNA fragments is a hallmark of cell apoptosis and is executed within the dying cells (autonomous) or in the engulfing cells (non-autonomous). The TUNEL (terminal deoxynucleotidyl transferase dUTP nick end labelling) method is used as an *in situ* assay of apoptosis by labelling DNA fragments generated by caspase-associated DNase (CAD), but not those by the downstream DNase II. In the present study, we report a method of ToLFP (topoisomerase ligation fluorescence probes) for directly visualizing DNA fragments generated by DNase II in *Caenorhabditis elegans* embryos. ToLFP analysis provided the first demonstration of a cell autonomous mode of DNase II activity in dying cells in *ced-1* embryos, which are defective in engulfing apoptotic bodies. Compared with the number of ToLFP signals between *ced-1* and wild-type (N2) embryos, a 30% increase in N2 embryos was found, suggesting that the ratio of non-autonomous and autonomous modes of DNase II was ~3–7. Among three DNase II mutant embryos (*nuc-1*, *crn-6* and *crn-7*), *nuc-1* embryos exhibited the least number of ToLFP. The ToLFP results confirmed the previous findings that NUC-1 is the major DNase II for degrading apoptotic DNA. To further elucidate NUC-1′s mode of action, *nuc-1*-rescuing transgenic worms that ectopically express free or membrane-bound forms of NUC-1 fusion proteins were utilized. ToLFP analyses revealed that anteriorly expressed NUC-1 digests apoptotic DNA in posterior blastomeres in a non-autonomous and secretion-dependent manner. Collectively, we demonstrate that the ToLFP method can be used to differentiate the locations of blastomeres where DNase II acts autonomously or non-autonomously in degrading apoptotic DNA.

## Abstract (precis)

The method of ToLFP (topoisomerase labelled fluorescence probes) is useful for detecting the DNA fragments generated by DNase II in *Caenorhabditis elegans* embryos. It reveals ~70% ToLFP signals in dying cells and 30% in engulfing cells during embryogenesis.

## INTRODUCTION

Programmed cell death (PCD) or apoptosis plays a vital role in animal development and tissue homoeostasis and is tightly regulated by a multitude of hydrolytic enzymes [1,2]. Subsequent to the activation of caspases, cells trigger a cascade of enzymes involved in digesting DNA of apoptotic bodies [3]. The early events of internucleosomal chromatin DNA fragmentation are mediated by a family of endonucleases called CAD (caspase-associated DNase) [4]. CAD is a magnesium-dependent endonuclease specific for cutting dsDNA to generate 3′-hydroxy ends [5], which is a hallmark of apoptosis. Accordingly, established assays for cell apoptosis entail detection of the appearance of DNA ladders (internucleosomal DNA) by agarose gels and signals of TUNEL (terminal deoxynucleotidyl transferase dUTP nick end labelling) by *in situ* extension of poly-dUTP from the 3′-hydroxy ends (Figure 1) [6,7].

**Figure 1:**
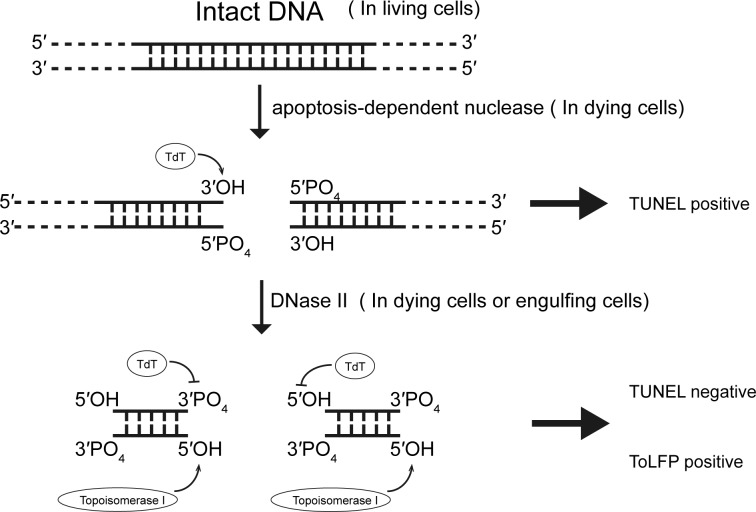
Illustration of *in situ* TUNEL assay in consecutive steps of DNA degradation during apoptosis During cell apoptosis, inside of dying cells CADs cleavage the intact DNA into fragments exposing 3′-hydroxy ends which can be extended with poly-dUTP by terminal deoxynucleotidy transferase (TdT) and shown as TUNEL positive. Subsequently, the DNA fragments are then digested by DNase II, either in dying cells or in engulfing cells, to yield products with 5′-hydroxy ends and 3′-phsophate ends which are unable to be reacted with tdt and shown as TUNEL negative. In the present study, a ToLFP was employed to directly label the 5′-hydroxy ends and shown as ToLFP positive.

The later steps of further degradation of fragmented DNA in cell apoptosis rely on DNase II (EC 3.1.22.1), an acidic deoxyribonuclease. Due to its lysosomal localization and optimal activity at pH 4.5–5.5, DNase II was initially thought to play a role in the digestion of exogenous DNA engulfed by phagocytosis in many animals [8,9]. Subsequent studies in *Caenorhabditis elegans* found that NUC-1 (a key member of three *C. elegans* DNase II enzymes) is also involved in DNA fragmentation and degradation during cell apoptosis [10]. DNase II is known to act downstream of CAD to further digest large DNA fragments into small DNA fragments or mononucleotides by hydrolysing the phosphodiester linkages in both native and denatured DNA to yield products with 5′-hydroxy ends and 3′-phosphate ends (Figure 1). As these structures are not substrates for terminal transferase [8,11,12], DNase II activity *in situ* is correlated with decreases in TUNEL signals, albeit indirectly.

In mammalian apoptosis, the action of DNase II is mostly restricted to the phagocytes or engulfing cells that digest DNA fragments of apoptotic cells and belongs to a unique class that exhibits cell non-autonomous activity [9,13–15]. Whether DNase II functions as a cell-autonomous nuclease remains controversial. However, the view that DNase II can function autonomously has been supported by two lines of evidence: (1) DNase II have been detected in the nuclei of Chinese hamster ovary (CHO) and HL-60 cells and (2) intracellular acidification occurs in many apoptotic cells [16,17]. It was suggested that DNase II could be released from lysosomes for cleaving nuclear DNA when the intracellular acidification occurs during cell apoptosis [16,18]. Furthermore, a cell autonomous action of DNase II has been found in *Drosophila*, through which the DNA of dying nurse cells under starvation-induced necrosis is degraded [19]. In *C. elegans*, the autonomous action has been demonstrated by the findings that DNA is digested into small pieces in the *ced-1* (encoding a transmembrane receptor for cell corpse recognition) mutant background [20]. In addition to the *ced-1* mutant, the TUNEL signals were increased in other engulfment-defective mutants (*ced-2*, *ced-5*,*ced-6* and *ced-10*) in the absence of NUC-1 [10,21], thus suggesting that the DNA fragments are normally digested within dying cells in an autonomous manner.

Previous reports have demonstrated that the functional roles of NUC-1 in PCD and engulfment-mediated DNA degradation in *C. elegans* comprises two phases: an autonomous action shown by a negative TUNEL staining in dying cells followed by a non-autonomous action of DNA elimination in phagocytic cells [10,11,20]. However, questions with regard to these modes of DNase II action in worms, particularly related to spatial manifestation and function representation, remain unanswered. In the present study, we employed a method, ToLFP (topoisomerase ligation of fluorescence probes), for directly labelling the DNA breaks generated by DNase II with fluorescence probes [22–25]. By applying ToLFP to examine worms of various genetic backgrounds, our current results show that the relative representation of the autonomous and non-autonomous actions of DNase II is ~70%–30% and further demonstrate that the ToLFP method can complement with the method of TUNEL in studying apoptotic DNA degradation.

## MATERIALS AND METHODS

### Strain maintenance

All strains were maintained with standard procedures and raised at 20°C [26]. Bristol N2 was used as a wild-type animal. Two apoptosis mutants [LGIV: *ced-3(n717)* and LGI: *ced-1(e1735)*] and three DNase II mutants: LGIII: *crn-6(tm890)*, *crn- 7(ok866)*; LGX: *nuc-1(e1392)* were described previously [21]. Three DNase II double mutants [*crn-7*(*ok886*) *crn-6*(*tm890*), *crn-7*(*ok866*); *nuc-1*(*e1392*) and *crn-6*(*tm890*); *nuc-1*(*e1392*)] and one triple mutant (*crn-7 crn-6*; *nuc-1*) were obtained from a previous study [21]. A transgenic worm of *smIs172* [*nuc-1* (*e1392*); *P_nuc-1_nuc-1::gfp*] was created previously [21] whereas another transgenic worm of *cguIs3* [*nuc-1* (*e1392*); *P_nuc-1_nuc-1::lmp-1_153–237_::gfp*] was generated in the present study.

### Generation of cguIs3 transgenic worm

Plasmid of p*P_nuc-1_nuc-1::lmp-1_153–237_::gfp* was constructed by insertion of a PCR fragment of *lmp-1_153–237_* into the MscI and AgeI restriction enzyme sites of p*P_nuc-1_nuc-1::gfp* and was confirmed by sequencing. The primers for amplifying the *lmp-1_153–237_* fragment were as follows: forward primer: CCAAtgctccaccgcca; reverse primer: TTCTACCGGTTTgacgctggcatatccttg. The integration line of *cguIs3* was obtained by microinjection with the target plasmid (100 μg/ml) and a plasmid with the *rol-6* selection marker (40 μg/ml) and following to use the UV/TMP method as described previously for selection of transgene integration [27]. The integration line was back-crossed by mating to *nuc-1*(*e1392*) males for four times.

### Topoisomerase ligation of fluorescence probe to detect the DNase II acting sites

ToLFP was applied to detect the DNA fragmentation that is specifically generated by DNase II in embryos. As previously described by Minchew and Didenko [22–25], a fluorescent probe was able to label the DNA fragments having 5′-hydroxy ends (DNase II-type breaks) by topoisomerase. ApopTag® ISOL Dual Fluorescence Apoptosis Detection Kit (APT1000) was purchased from Merck Millipore. We followed the standard protocol that was provided by Merck Millipore with a slight modification. Fixation of embryos was conducted as previously described [28]. Fixed samples were added into a solution that was premixed with topoisomerase and fluorescent probes. After 12–16 h of incubation at 20°C, embryos were washed three times with PBST (1× PBS with 0.1% Tween 20) and then mounted on to a slide which contains 2% agar pad. Z-stack images were detected and acquired under a fluorescence microscope (Leica DM IRE2 with BD CARV II) with a FITC filter.

### *In vitro* assay of DNase II activity

DNase II activities of embryo lysates were analysed by gel electrophoresis. Embryos were harvested by hypochlorite/NaOH treatment and lysed by a sonication method in PBST buffer containing protease inhibitors (Roche). Protein quantification was performed by measurement of *A*_595_ using a Dye Reagent Concentrate (Bio-Rad). One microgram of each embryo extract was incubated with 25 μg of salmon sperm DNA (Sigma) at 37°C in 100 μl of DNase II assay buffer (100 mM sodium acetate, pH 4.5, 10 mM EDTA) and collected at desired time point. Samples were separated by 1% agarose gel and stained with EtBr (ethidium bromide) for determination of DNase II activity.

### Metachromatic agar diffusion assay

DNase II activities of embryo lysates were also examined by metachromatic agar-diffusion assay (MADA), as previously described [29] with some modifications. The boiled 1% agarose gel contained DNase II assay buffer (100 mM sodium acetate, pH 4.5, 10 mM EDTA) and mixed with salmon sperm DNA (40 μg/ml; Sigma) and EtBr (400 μg/ml) at 65–70°C, was then poured into a 9-cm plastic petridish. The wells were made on solidified gels with a Pasteur pipettes using 5 μg of samples, as determined by the measurement of an *A*_595_, where the amount of tubulin was verified by Western blotting as a loading control. Crude extracts were prepared from embryos, which were collected by hypochlorite/NaOH treatment and homogenized in PBST (by douncing 20 times with pre-cooled Dura-Grind stainless steel dounce tissue grinder (Wheaton). After 16-h incubation at 37°C, a ring like DNA consumption pattern was observed under UV light, which represents the DNase II activity for each sample. The area of dark ring was measured by ImageJ software. A DNase II activity standard was included employing porcine DNase II (Sigma) in 0.5–50 KUs (Kunitz units).

### Western blotting

Total proteins from various genetic backgrounds (N2, *ced-3*, *ced-1*, *smIs172* and *cguIs3*) embryos were separated by SDS/gel electrophoresis and processed for Western blot analysis. Five micrograms of each embryo extract was added into 1× sample buffer in a 20 μl of total volume and boiled at 100°C for 15 min. The lysate was cooled on ice for 5 min and then centrifuged to remove debris and stored at −80°C. Anti-actin (1:100000) was used as an internal control of loaded protein for the *in vitro* DNase II activity assay.

### Immunofluorescence of staining ER-marker and GFP

Embryos from *smIs172* and *cguIs3* were collected by hypochlorite/NaOH treatment. After freeze cracking, embryos were fixed with 3.7% formaldehyde, 75% methanol, 0.5× PBS for 15 min at −20°C followed by 15 min in 100% methanol and then incubated in a blocking solution (30% goat serum in PBS/0.1% Tween 20) for 1 h at room temperature (RT). The primary antibodies of anti-GFP (GeneTex) and anti-HDEL (tetrapeptide of His–Asp–Glu–Leu) antibody (Santa Cruz) with 1:200 dilution in blocking buffer were reacted with embryos for 12–16 h at 4°C, the secondary antibodies anti-rabbit AlexFluor-488 (for staining GFP) and anti-mouse AlexFluor-594 (for staining ER, endoreticulum; Invitrogen, 1:500) were reacted for 2–4 h at RT, following by 5 min DAPI staining. Samples were washed three times with PBST after each step of reaction. Images were taken using Zeiss Axio Imager.Z2 with apotome 2.

### STYO 11 staining

The undigested bacteria DNA in the gut lumen was stained by vital DNA-binding dye SYTO11 (Molecular Probe, Invitrogen) as previously described [21]. The L3 and L4 stage worms were collected and washed twice with M9 buffer then transferred into a 1.5 ml of tube containing SYTO11 dye (10 μM in M9) at RT for 2 h in the dark. The stained worms were washed twice with M9 and recovered on *Escherichia coli* OP50 seeded NGM (nematode growth medium) plates at RT for 1 h in the dark. After recovery, the worms were washed twice with M9 and anaesthetized by lavamisole (200 μM), then mounted on to 2% agar pads and observed under a fluorescence microscope (Leica DM2500) with a FITC filter.

## RESULTS

### ToLFP is useful in differentiating blastomeres for DNase II activities in an autonomous or non-autonomous manner

Since the TUNEL method is an indirect measurement of the DNase II activity *in vivo*, it is hardly to distinguish the autonomous and non-autonomous action of DNase II in *C. elegans*. We adapted a newly established method called ToLFP to directly label the 5′-hydroxy ends of DNA fragments, which are the products of DNase II digestion, with the FITC-labelled DNA fragments [22]. To distinguish the autonomous and non-autonomous action of DNase II, we first examined ToLFP signals in the wild-type worm (N2) embryos and two mutant embryos of *ced-3* (encoding a homologue of mammalian caspase) and *ced-1* (encoding a transmembrane receptor for cell corpse recognition). As compared with positive signals of ToLFP in N2 embryos, no ToLFP signal was observed in *ced-3* embryos, whereas significant numbers of ToLFP signals were observed in *ced-1* embryos at the comma stage (Figure 2A). By contrast, similar levels of DNase II activities were observed in N2, *ced-3* and *ced-1* embryo lysates when the lysates were assayed *in vitro* under the condition of pH 4.5 (Figure 2B). The differential detection of DNase II activity of *ced-3* embryos between *in vitro* assay and *in vivo* assay (Figure 2B, lanes 7–9 compared with Figure 2A, frame c) was probably attributed to the lack of CED-3 (caspase) in worms, which leads to no apoptosis and DNA fragmentation, but the presence of functional DNase II enzyme. Furthermore, the quantitative difference of ToLFP numbers between N2 and *ced-1*embryos (Figure 2A frame b compared with frame d) arose from defective apoptotic body engulfment in *ced-1* embryos. The ToLFP signals from *ced-1* embryos were presumably due to the autonomous action of DNase II only.

**Figure 2:**
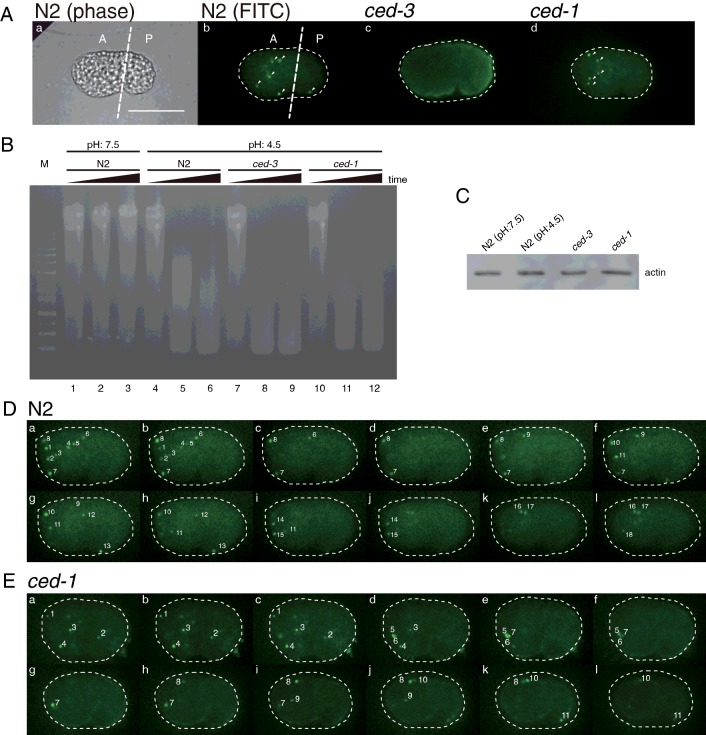
ToLFP staining in embryos from wild-type animals (N2) and two apoptosis related mutants, *ced-3* (caspase defective mutant) and *ced-1* (a defective mutant of engulfing apoptotic bodies) (**A**) A representative image of ToLFP labelling patterns from N2, *ced-3* and *ced-1* embryos. The same embryo of N2 is shown in a phase image (**a**) and fluorescence image (**b**), a *ced-3* embryo in a fluorescence image (**c**) and a *ced-1* embryo in a fluorescence image (**d**). The imaginary line in (**a**) and (**b**) indicates anterior (A) and posterior (P) half of embryo. Scale bar: 20 μm. (**B**) An *in vitro* assay of DNase II activity. Embryo extracts from N2, *ced-3* and *ced-1* were analysed the DNase II activity under the condition of pH 7.5 (lane 1–3) or pH 4.5 (lane 4–12). The samples of each strain were collected and analysed at three time points: 0, 15 and 30 min. The lysates from various genetic background worms are indicated above the gel. M lane shows DNA ladder markers. (**C**) The Western blot analysis of actin is used as loading amount of lysates. (**D**) Quantitative analysis of ToLFP number of whole embryos. A series section of images (a–l) from a single N2 embryo shows the total ToLFP signals (labelled with numbers). (**E**) A series section of images (a–l) from a single *ced-1* embryo shows the total ToLFP signals (labelled with numbers). Scale bar: 20 μm.

To quantify the total ToLFP number in each embryo from N2 and *ced-1* mutants, we photographed each individual embryo under a fluorescence microscope with Z-axis sections. Representative results from N2 and *ced-1* embryos are shown in Figures 2(D) and 2(E) respectively. Approximately 14 of ToLFP signals were observed in the *ced-1* embryos (Table 1) clearly illustrating DNase II activity in the dying cells (autonomous action) but lack thereof in the engulfing cells (non-autonomous action). Since only part of embryonic cells expresses NUC-1 and given that not all the cells destined to die autonomously express NUC-1, the six additional ToLFP signals in N2 embryos in comparison with the *ced-1* embryos were suggested to be from non-autonomous action (Table 1). As a whole embryo, the percentage of non-autonomous to autonomous degradation of apoptotic DNA was ~30%–70%; however, a higher percentage (~45%) of non-autonomous degradation of DNA was found in the posterior half of embryos (Table 1). It was probably a result of higher level of NUC-1 expression in the anterior half of embryos and subsequent secretion to and uptake by the posterior cells of embryos.

**Table 1:** The summarized data of autonomous and non-autonomous action of DNase II in C. *elegans* through ToLFP staining Embryos were scored at the 1.5-fold stage. The data shown are mean ± S.E.M. N-number: N2=13 and *ced-1(e1735)*=*12*.

			Percentage of apoptotic DNA degradation by autonomous and non-autonomous manners	
	Genotype	Number of ToLFP	Autonomous	Non-autonomous
Whole embryo				
	N2	19.85±0.7	70% (13.92/19.85)	30% (5.93/19.85)
	*ced-1*	13.92+0.6	100% (13.92/13.92)	0% (0/13.92)
Head				
	N2	16.54+0.7	74% (12.17/16.54)	26% (4.37/16.54)
	*ced-1*	12.17+0.6	100% (12.17/12.17)	0% (0/12.17)
Tail				
	N2	3.3+0.3	55% (1.8/3.3)	45% (1.5/3.3)
	*ced-1*	1.8+0.2	100% (3.3/3.3)	0% (0/3.3)

### ToLFP analysis is consistent with TUNEL analysis for DNA degradation in three DNase II mutants

To support the above results and to confirm our previous TUNEL study showing that NUC-1 plays the major role of DNase II and CRN-6 is auxiliary to NUC-1 in the apoptosis during the early embryonic development [21], we then compared ToLFP signals in three strains of DNase II mutant embryos (*nuc-1*, *crn-6* and *crn-7*) with the wild-type (N2) embryos at the comma, 1.5-fold and 2-fold stages. As shown in Figure 3(A), *nuc-1* embryos exhibited the least number of ToLFP signals among the four strains. However, the average size of the ToLFP signal was the largest in *nuc-1* embryos (Figure 3B), suggesting a distinct mode of action of NUC-1 from that of CRN-6 or CRN-7. To further quantify the total ToLFP-foci numbers in all examined embryos, we photographed each individual embryo under a fluorescence microscope with Z-axis sections as described above and compiled each image into a movie (Supplementary movies S1–S12). The quantitative data of total ToLFP signals from at least 15 embryos are summarized in Figure 3(C), which showed that both N2 and *crn-7* had on average 19–20 labelled signals in contrast with 7 and 14 exhibited by *nuc-1* and *crn-6* respectively. These findings were consistent with the *in vitro* MADA analyses that NUC-1 is the primary DNase II for the clearance of apoptotic DNA in dying cells during embryogenesis (Figure 4). The semi-quantitative data of MADA were summarized from three independent experiments in which the N2 activity was regarded as 100% (Figure 4C). The cell extracts from embryos of double mutants, *crn-7*(*ok886*) *crn-6*(*tm890*), *crn-7*(*ok866*); *nuc-1*(*e1392*) and *crn-6*(*tm890*); *nuc-1*(*e1392*) respectively, showed 90.6%, 1.4% and 3.6% of the total DNase II activity of N2 (Figure 4C), indicating that NUC-1 alone had 30–50 folds higher activity than either CRN-6 or CRN-7. This conclusion was further supported by the significantly reduced DNase II activity (7.5% of total) in the *nuc-1* mutant, which presumably was contributed by CRN-6 and CRN-7.

**Figure 3:**
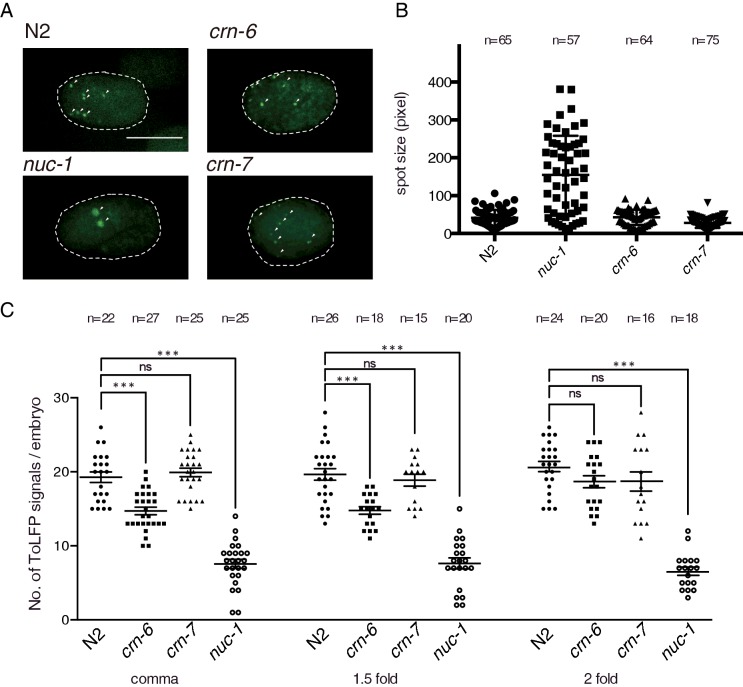
ToLFP assay of embryos from N2 and three DNase II mutants (**A**) ToLFP labelling patterns of N2, *nuc-1*, *crn-6* and *crn-7* embryos at the 2-fold stage. Green spots are ToLFP positive signals, which are indicated with white arrows. The anterior of the embryo is shown towards the left. Scale bar: 20 μm. (**B**) The spot size of ToLFP signals in DNase II mutants. The spot sizes were determined by ImageJ software. More than 10 embryos and over 50 signals were scored for each strain at the comma stage. (**C**) Quantitative analysis of ToLFP signals in three DNase II mutants at the comma, 1.5-fold and 2-fold stages. At least 16 embryos were scored for each stage and strain. Error bars indicate S.E.M., ***P > 0.001. ns, non-significant.

**Figure 4:**
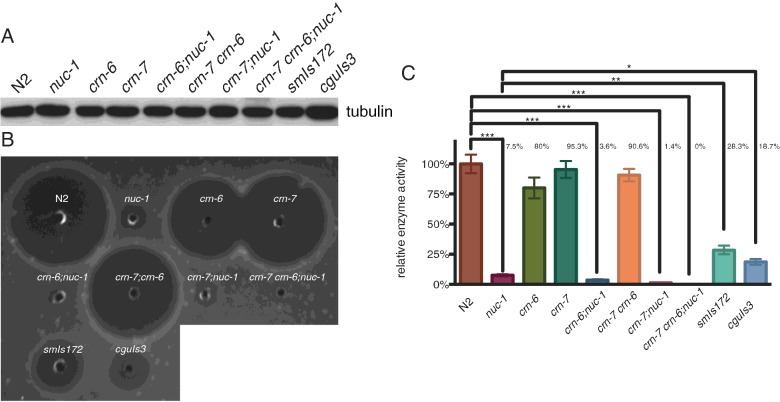
Analysis of DNase II enzymatic activity of embryo lysate in various genetic backgrounds as indicated by MADA (**A**) Western blotting with anti-tubulin antibody (in 1:5000 dilution) to show a similar amount of each lysate was loaded. (**B**) A representative MADA result showing DNase II enzymatic activity. (**C**) Quantitative analysis was determined by the area of the clear zone on agarose from three independent experiments. The enzymatic activity of DNase II in N2 is served as 100% and the relative percentage of DNase II activity from each strain of worms is indicated. *N*=3, Error bars indicate S.E.M., *P > 0.05, **P > 0.01, ***P > 0.001.

### Phenotypic characterization of two transgenic lines expressing different NUC-1 fusion proteins

Since *nuc-1* mutants showed the least ToLFP signals, an increasing number of ToLFP signals in transgenic embryos which express various forms of NUC-1 fusion proteins can be easily detectable. We then employed two transgenic lines, *smIs172*[*nuc-1* (*e1392*); *P_nuc-1_nuc-1::gfp*] and *cguIs3*[*nuc-1* (*e1392*); *P_nuc-1_nuc-1::lmp-1*_153–237_*::gfp*] to examine their capability of rescuing of *nuc-1* mutant phenotypes and the distribution of ToLFP signals in embryos. Transgenic worms of *smIs172* express NUC-1::GFP fusion protein whereas *cguIs3* worms express a different version of fusion protein (NUC-1::LMP-1_153–237_::GFP) with the insertion of the membrane domain of lysosomal protein LMP-1 between NUC-1 and GFP. This additional transmembrane sequence is known to promote recruitment into lysosomal membrane with the C-terminal portion facing the cytoplasm [30]. The results of MADA showed that lysates from *smIs172* and *cguIs3* had lower DNase II activity in comparison with N2 (respectively 28.3% and 18.7%), but higher activity than that of *nuc-1* mutant (7.5%; Figure 4C), suggesting that the fusion proteins are functional but slightly compromised by the addition of GFP. A lower DNase II activity of *cguIs3* than that of *smIs172*, as well as the observation that both transgenic worms could not exert the full activity of DNase II as N2 worms are probably due to additional GFP and lysosomal segments that disrupt the NUC-1′s proper folding. Nevertheless, double staining of anti-GFP and anti-HDEL (ER marker) in *smIs172* and *cguIs3* transgenic worms revealed that both NUC-1 fusion proteins appeared at the anterior blastomeres of embryos and were co-localized with ER with a slightly different pattern (Figure 5).

**Figure 5:**
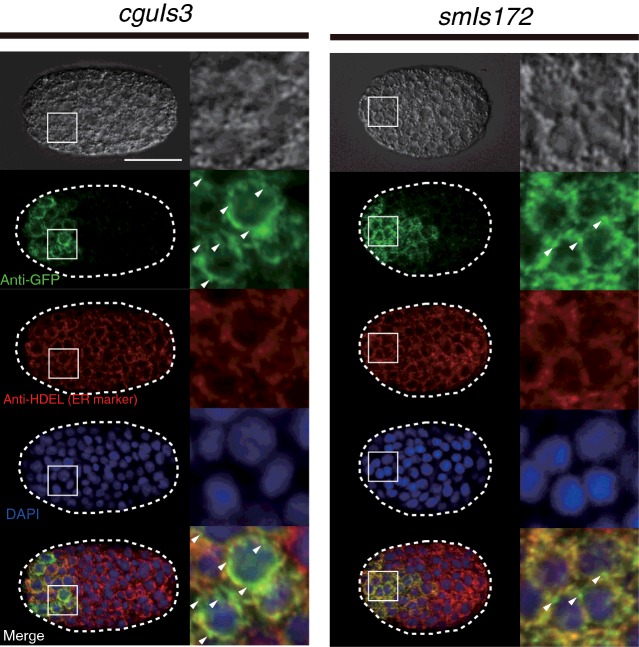
Characterization of the two NUC-1 fusion proteins in transgenic animals of *smIs172* and *cguIs3* The subcellular localization of NUC-1 fusion proteins in *smIs172* and *cguIs3* embryos. Immunostaining with antibodies against GFP (in 1:500 dilution) and against HDEL (ER marker, in 1:200 dilution) were performed on embryos of *smIs172* and *cguIs3*. Images are indicated with conditions of microscopy for photography. Nucleus was labelled with DAPI. The anterior of embryo is shown toward left and those cells located in the anterior region are enlarged to show co-localization of NUC-1 fusion proteins with ER proteins portion in detail. Imperfect merged of GFP and ER markers are indicated by arrows. Scale bar: 20 μm.

### Two NUC-1 fusion proteins are biologically functional in digesting bacterial DNA but varied in non-autonomous degradation of apoptotic DNA

After having obtained MADA results showing a partial recovery of NUC-1 activity by NUC-1::GFP and NUC-1::LMP-1_153–237_::GFP fusion proteins (Figure 4C), we then examined if the fusion proteins in two transgenic worms can rescue the *nuc-1* phenotypes. Four strains of worms (N2, *nuc-1*, *smIs172* and *cguIs3*) were stained with DNA dye and then examined for the presence of bacterial DNA in the intestinal lumen under a fluorescence microscope. In contrast with 90% positive DNA staining in the intestine lumen of *nuc-1* worms, 10% and 12% of DNA staining were found in the *smIs172* and *cguIs3* worms respectively, with 7% positive staining in wild-type worms (N2; Figure 6). Alleviation of the defective phenotype by the ectopic NUC-1::GFP and NUC-1::LMP-1_153–237_::GFP indicated that they are functional counterpart of the wild-type protein.

**Figure 6:**
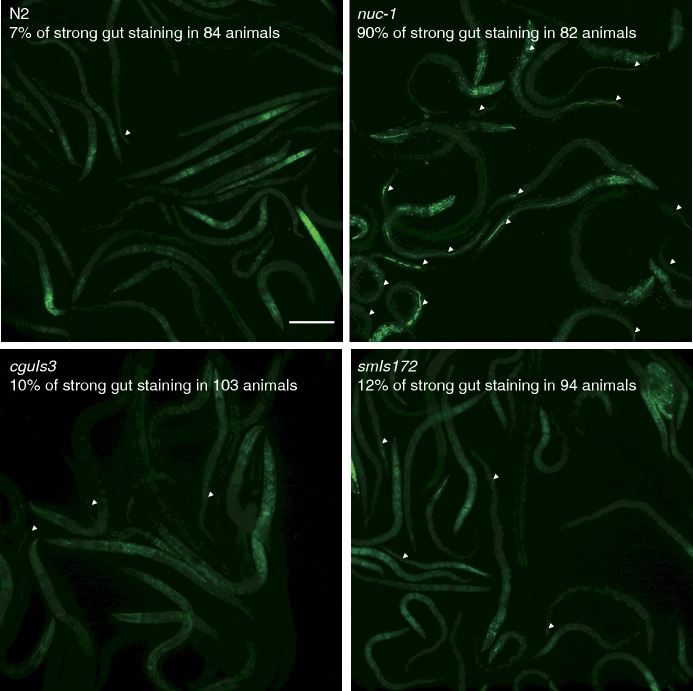
Functional assays for the two NUC-1 fusion proteins in digesting bacterial DNA in the gut Staining images of undigested bacteria DNA in the gut lumen by SYTO11. The positive signals are indicated with white arrowheads. The number of scored animals and the percentage of positive DNA staining are shown beneath the strain name in each frame. Scale bar: 100 μm.

Next, we applied ToLFP to examine whether these two NUC-1 fusion proteins could remove apoptotic DNA in transgenic embryos during embryogenesis. To quantify the number of ToLFP signal from embryos of *nuc-1*, *smIs172* and *cguIs3* at the comma, 1.5-fold and 2-fold stages, we performed Z-axis sectioning photography as described in Figure 3. Data from at least 18 embryos showed that *nuc-1* embryos exhibited approximately six signals at each stage, whereas numbers of ToLFP signals were elevated to 13 and 10 respectively, in *smIs172* and *cguIs3* embryos (Figure 7A), indicating a higher extent of DNA digestion in the presence of NUC-1::GFP and NUC-1:: LMP-1_153–237_::GFP. However, the increased number of ToLFP in *smIs172* was also significantly higher than that in *cguIs3*, suggesting that the two fusion proteins function in different modes or with different levels of activity. Since NUC-1 is expressed in the anterior portion (head) of embryos, we next sought to evaluate any non-autonomous action of the two NUC-1fusion proteins in the posterior (tail) of embryos. The counts of ToLFP signals in the embryos were grouped according to spatial distribution (head or tail) for the *nuc-1*, *smIs172* and *cguIs3* strains. Such analysis showed that whereas ToLFP signals remained significantly elevated in the head region of *smIs172* and *cguIs3* embryos as compared with *nuc-1* (Figure 7B), they dropped considerably in the tail of *cguIs3* embryos in all three stages examined (Figure 7C). Conversely for the *smIs172* embryos, significantly increased ToLFP signals were observed in the tail at the comma and 1.5-fold stages, but not at the 2-fold stage. Together, these data revealed that NUC-1::GFP, but not NUC-1:: LMP-1_153–237_::GFP, could execute degradation of apoptotic DNA non-autonomously in the tail cells of embryos. We reasoned that the difference might be contributed by the presence of membrane domain of LMP-1 in the protein of NUC-1::LMP-1_153–237_::GFP, which blocks the secretion of this protein.

**Figure 7:**
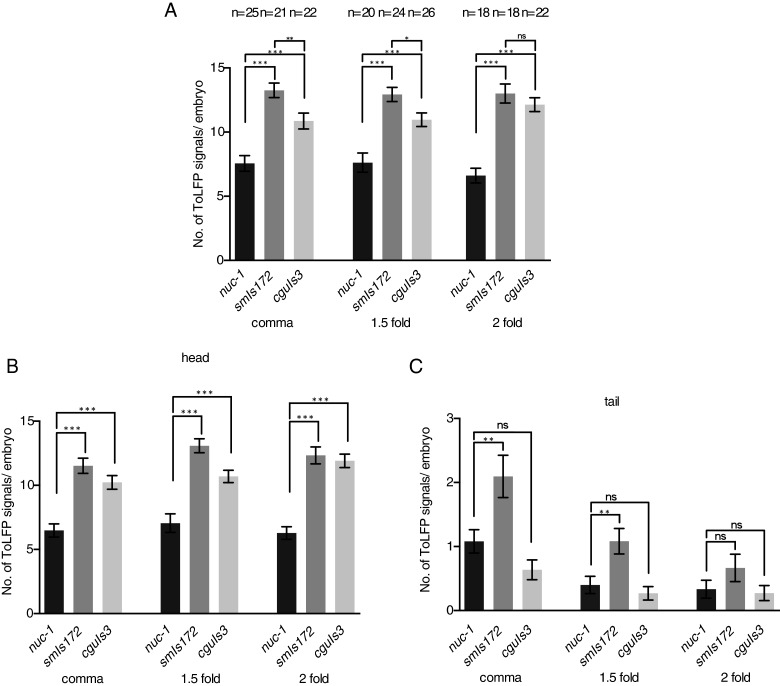
Quantitative analysis of ToLFP signals shows the rescue of *nuc-1* phenotypes by two transgenes in *smIs172* and *cguIs3* animals (**A**) The ToLFP signals of *nuc-1* mutants and transgenic worms in whole embryos at the comma, 1.5-fold and 2-fold stages. (**B**) The ToLFP signals of *nuc-1* mutants and transgenic worms in the head region of embryo at the comma, 1.5-fold and 2-fold stages. (**C**) The ToLFP signals of *nuc-1* mutants and transgenic worms in the tail region of embryo at the comma, 1.5-fold and 2-fold stages. The n number of executed embryos in each strain and stage is indicated above the bar. At least 18 embryos were scored for each stage and strain. Error bars indicate S.E.M., *P > 0.05, **P > 0.01, ***P > 0.001. ns, non-significant.

## DISCUSSION

In the past, most reports on detecting DNase II activity of various animal samples were based on *in vitro* assays [29,31–33] and only in a few papers from Didenko's laboratory were assays done *in vivo* [22–25]. In the present study, we employed a newly established *in vivo* method of ToLFP to directly illustrate DNA breaks generated by DNase II in the embryonic cells, for which TUNEL was previously used for indirect observation. In addition, in combination of various genetic backgrounds of worms, the ToLFP method can differentiate the autonomous or non-autonomous action of DNase II in the embryonic cells and confirms that NUC-1 is principally responsible for degradation of apoptotic DNA during embryogenesis (Figures 3 and 4). Importantly, ToLFP showed for the first time that the autonomous degradation of apoptotic DNA, presumably contributed by NUC-1 and CRN-6, occurs in *ced-1* embryos at the comma stage (Figures 2D compared with 2E; Table 1). Furthermore, since the ToLFP signals in *nuc-1* mutant embryos represent the functional sites of CRN-6 and/or CRN-7, their distribution in the precursor of intestine cells evidence the probably non-autonomous action of CRN-6. The larger size of ToLFP signals present in the *nuc-1* mutant, indicative of greater accumulation of DNA fragments detected by fluorescent probes (Figures 3A and 3B), also implies that NUC-1 acts differently from CRN-6 and CRN-7, the less active enzymes. Whether the different sizes of ToLFP signal represent various stages of apoptotic body, similarly to those reported in germ cell apoptosis stained by the DNA dye [34], remains unknown. Elucidating how NUC-1 acts more actively than CRN-6 and CRN-7 awaits the solution of the 3D structure of each DNase II.

Further ToLFP studies of *smIs172*, but not *cguIs3*, showed a significantly higher number of ToLFP signals in the tail region of embryos, confirming that NUC-1 *in vivo* can act non-autonomously to remove apoptotic DNA in the tail region embryos at specific stages (Figure 7). The appearance of ToLFP in the tail of embryos of the double mutant of *crn-6* and *crn-7*, which expresses NUC-1 only, also supports this supposition (Supplementary Figure S1). The two transgenic worms also provided a new line of evidence that fusion proteins of NUC-1 are enzymatically functional (Figures 4B and 4C). However, both transgenic worms showed a similar capability in digesting bacteria DNA in the intestine lumen (Figure 6) but *cguIs3* worms appeared less efficiently in removing apoptotic DNA in embryos (Figure 7). Accordingly, a hypothetical model shown in Figure 8 depicts the maturation pathways of two fusion proteins and their possible distinct actions on the clearance of bacterial DNA and apoptotic DNA. In this model, both NUC-1 fusion proteins complete the biosynthesis in ER then either enter a secretory pathway or become localized to lysosomes, in response to bacterial DNA or apoptotic signalling. In this context, the NUC-1::GFP fusion protein is probably released into the lumen of intestine for degrading bacterial DNA or the extracellular space followed by engulfment into neighbouring cells for cleaving apoptotic DNA non-autonomously (Figures 8A and 8B). Whereas NUC-1::LMP-1_153–237_::GFP may follow a similar fate (Figures 8C and 8D), upon trafficking it probably remains on the plasma membrane with the DNase II domain facing the intestine lumen, thus retaining activity towards bacterial DNA but not its non-autonomous action in the neighbouring cells.

**Figure 8:**
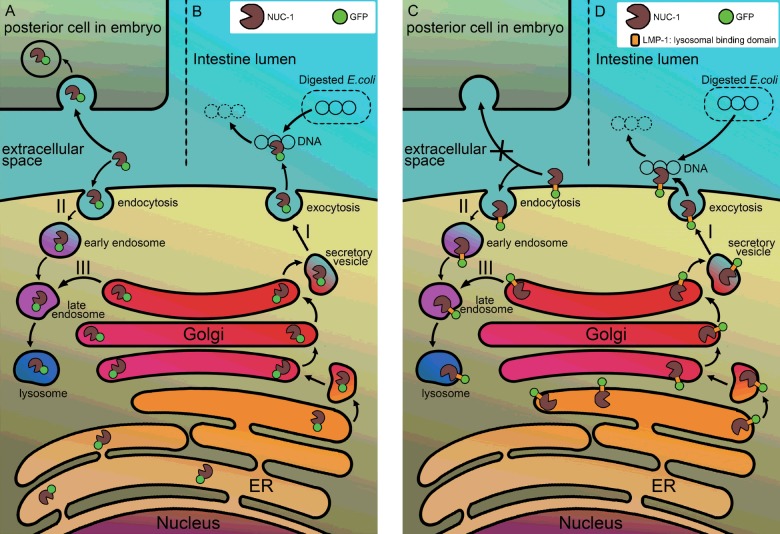
A hypothetical model illustrates the maturation pathway and action mode of two NUC-1 fusion proteins The N-terminal leader sequence present in the NUC-1 of fusion proteins leads the polypeptide to ER for completion of biosynthesis. The NUC-1::GFP protein is then released into the cisternae of ER as a free form of protein in *smIs172* animals (left panel). The additional LMP-1 sequence present in the NUC-1::LMP-1_153–237_::GFP protein renders the fusion protein appearing as a transmembrane protein, in which the GFP is located outside of ER and the DNase II domain is facing to ER lumen (right panel). Most of fusion proteins are resided in ER, upon signalling of bacterial DNA or apoptosis, both move to Golgi apparatus for further glycosylation and being secreted through vesicles transportation to the plasma membrane. After the vesicles are fused with plasma membrane, the NUC-1::GFP fusion protein is released into the lumen of intestine for degradation of bacterial DNA (**B**) or into the extracellular space and then being retaken up by neighbouring cells (**A**) for degradation of apoptotic DNA (pathway I). The secreted fusion protein can also go through endocytosis mechanism back to lysosomes of cells (pathway II). Whereas the NUC-1::LMP-1_153–237_::GFP protein remains on the plasma membrane of intestine cells or embryonic blastomeres (**D**) (pathway I), it is able to digest bacterial DNA but unable to be retaken up by posterior cells for degradation of apoptotic DNA non-autonomously (**C**). However, it can be recycled through endocytosis pathway to target to lysosomes (pathway II). Both fusion proteins have a regular pathway targeting to lysosomes (pathway III). Symbols of fusion proteins are indicated by green circle as GFP and a pacman as DNase II.

In addition, this notion further hints that NUC-1::LMP-1_153–237_::GFP can function as a membrane bound form and in a single-polypeptide form. Many reports support the possibility that DNase II can function without any further cleavage into smaller polypeptides [35–37]. However, a recent paper indicated that cathepsin L is responsible for cleaving mammalian DNase II into smaller fragments inside of lysosomes [32]. It remains unknown whether the processed DNase II has up-regulated enzyme activity. We speculate that activation of DNase II may require further cleavage by cathespin L in *C. elegans*, since the mutation of *clp-1* (the homologue of cathespin L) prolongs the timing of removing the germ cell corpses [38], similarly to what we observed in *crn-7* mutants (Hsiang Yu and Szecheng J. Lo, unpublished data). To test whether a higher DNase II activity is achieved by CLP-1 cleavage in the lysosome, RNAi knockdown experiments of *clp-1* can be done to verify the hypothesis.

In summary, we employed the newly established method of ToLFP to illustrate the sites of the autonomous or non-autonomous action of DNase II in the embryonic cells and which DNase II is principally responsible for degradation of apoptotic DNA during embryogenesis. We suggest that the ToLFP method can complement the TUNEL assay in studying apoptotic DNA degradation. Since there are three waves of apoptosis occurring at the embryonic and the first larval stages and the germ cells of gonad in adults of *C. elegans* [39], we anticipate that this method can be further used to extend our knowledge of the roles and mechanisms of DNase II activity in the third wave of apoptosis in *C. elegans* germ cells. This useful method can be applied to other parasitic nematodes and invertebrates [19,35], in which both autonomous and non-autonomous actions of DNase II have been demonstrated.

## Abbreviations

CAD: caspase-associated DNase
ER: endoreticulum
EtBr: ethidium bromide
HDEL: tetrapeptide of His–Asp–Glu–Leu
MADA: metachromatic agar-diffusion assay
NGM: nematode growth medium
PCD: programmed cell death
RT: room temperature
ToLFP: topoisomerase ligation fluorescence probes
TUNEL: terminal deoxynucleotidyl transferase dUTP nick end labelling
